# 

**Published:** 1929

**Authors:** 


### The Bristol Medico-Chirurgical Journal

A JOURNAL OF THE MEDICAL SCIENCES FOR THE
WEST OF ENGLAND AND SOUTH WALES.

PUBLISHED UNDER THE AUSPICES OF
*THE BRISTOL MEDICO-CHIRURGICAL SOCIETY.*

EDITOR:
J. A. NIXON, C.M.G., M.D., F.R.C.P.

WITH WHOM ARE ASSOCIATED
E. W. HEY GROVES, M.S., F.R.C.S., B.Sc.
E. WATSON-WILLIAMS, M.C., Ch.M., F.R.C.S.E.
A. E. ILES, O.B.E., M.B., B.S. Lond., F.R.C.S.
CAREY F. COOMBS, M.D., F.R.C.P., Assistant Editor.

EDITORIAL SECRETARY:
A. L. FLEMMING, M.B., B.Ch.

---

"Scire est nescire, nisi id me
Scire alius sciret."

---

*VOL. XLVI.*

BRISTOL: J. W. ARROWSMITH LTD.
London: J. W. Arrowsmith (London) Ltd.

[illegible] MEDICAL LIBRARY

296938

WASHINGTON, D. C.

WI
BR323

W1 BR323 v46 1929 Fol. cont between 52-53

### The Medical Library of the University of Bristol,

WITH WHICH IS INCORPORATED

### The Library of the Bristol Medico-Chirurgical Society.

---

*The following donations have been received since the publication of the List in December,* 1928.

March, 1929.

| | |
|---|---|
| Librairie G. Doin et Cie, Paris (1) .. .. | 1 volume |
| The Eugenics Society (2) .. .. .. | 1 ,, |
| L'Université de Genève (3) .. .. .. | 5 pamphlets |
| Dr. V. B. Green-Armytage (4) .. .. | 2 volumes |
| The Dr. Griffiths Bequest (5) .. .. | 1 volume |
| Professor E. W. Hey Groves, M.S. (6) .. | 14 volumes |
| The London School of Hygiene and Tropical Medicine (7).. .. .. .. .. | 1 volume |
| The Michell Clarke Memorial Fund (8) .. | 34 volumes |
| The Munro Smith Memorial Fund (9) .. | 2 ,, |
| The Registrar (10) .. .. .. .. | 2 ,, |
| The Rockefeller Foundation (11) .. .. | 1 volume |
| The Smithsonian Institution (12) .. .. | 1 pamphlet |
| The Surgeon-General of the U.S. Army (13) | 1 volume |

Unbound periodicals have also been received from Dr. Carey Coombs, Dr. C. Corfield and Professor E. W. Hey Groves.

### THE ONE HUNDRED AND TWENTY-SEVENTH LIST OF BOOKS.

The figures in round brackets refer to the figures after the names of the donors. The books to which no such figures are attached have either been bought from the Library Fund or received through the *Journal.*

| | | | | |
|---|---|---|---|---|
| **Aldrich, L. B.** | *A Study of Body Radiation* | | (12) | 1928 |
| **Beaumont, G. E., and Dodds, E. C.** | *Recent Advances in Medicine* | 4th Ed. | (8) | 1928 |
| **Berry, R. J. A.** | *Brain and Mind* | | (8) | 1928 |
| **Binnie, J. F.** | *Manual of Operative Surgery* | 8th Ed. | (8) | 1921 |
| **Blair, V. P., and Ivy, R. H.** | *Essentials of Oral Surgery* | | (8) | 1925 |
| **Blondel, C.** | *The Troubled Conscience* | | | 1928 |
| **Brown, A.** | *Old Masterpieces in Surgery* | | (6) | 1928 |
| **Brown, A. J.** | *Dental Metallurgy* | | | 1928 |
| **Brown, W. L.** | *Endocrines in General Medicine* | | (8) | 1927 |
| **Buxton, P. A.** | *Researches in Polynesia and Melanesia.* Parts V.–VIII. | | (7) | 1928 |
| **Cabot, R. C.** | *Physical Diagnosis* | 9th Ed. | (8) | 1927 |
| **Cade, S.** | *Radium Treatment of Cancer* | | (6) | 1929 |

**Calmette, A.** .. .. *La vaccination préventive contre la tuberculose* (8) 1927
**Cameron, A. T.** .. .. *Textbook of Bio-Chemistry* .. .. .. .. (9) 1928
**Cameron, H. C.** .. *Reminiscences of Lister* .. .. .. .. .. .. .. 1927
**Carter, A. H.** .. .. *Elements of Practical Medicine* .. 11th Ed. (8) 1920
*Catalogue of the Library of the Ophthalmological Society of the United Kingdom* .. .. .. .. .. .. .. .. .. .. .. .. .. 3rd Ed. 1899
**Cassie, E.** .. .. .. .. *Maternity and Child Welfare* .. .. .. .. .. 1929
**Chandler, F. G., and Wood, W. B.** *Lipiodol in the Diagnosis of Thoracic Disease* .. .. .. .. .. .. .. .. .. .. .. .. .. 1928
**Cole, S. W.** .. .. *Practical Physiological Chemistry* .. 8th Ed. (9) 1928
**Colyer, J. F.** .. .. *Dental Surgery and Pathology* .. 5th Ed. (8) 1923
**Critchley, M.** .. .. *Mirror-writing* .. .. .. .. .. .. .. .. .. .. .. 1928
**Cushny, A. R.** .. .. *Textbook of Pharmacology and Therapeutics* 9th Ed. (8) 1928
**Dally, J. F. H.** .. .. *Low Blood Pressure* .. .. .. .. .. .. .. 1928
**Darwin, L.** .. .. .. *What is Eugenics?* .. .. .. .. .. .. .. .. (2) 1928
**Duke-Elder, W. S.** .. *Recent Advances in Ophthalmology* .. .. (8) 1927
**Duke-Elder, W. S.** .. *Practice of Refraction* .. .. .. .. .. .. .. (8) 1928
**Economo, C. von** .. *The Cytoarchitectonics of the Human Cerebral Cortex* 1929
**Eden, T. W., and Lockyer, C.** *Gynæcology* .. .. .. .. .. 3rd Ed. (8) 1928
**El-Eshary, N.** .. .. *La Cholecystographie* .. .. .. .. .. .. .. (3) 1927
**Ewing, J.** .. .. .. *Neoplastic Diseases* .. .. .. .. .. 3rd Ed. (8) 1928
**Fisher, A. G. T.** .. *Chronic (non-tuberculous) Arthritis* .. .. (6) 1928
**Flückiger, F. A., and Hanbury, D.** *Pharmacographia* .. 2nd Ed. (5) 1879
**Forrester-Brown, M. F.** *Diagnosis and Treatment of Deformities in Infancy and Early Childhood* .. .. .. .. .. .. .. .. .. 1929
**Fowweather, F. S.** .. *Handbook of Clinical Chemical Pathology* .. (6) 1929
**French, H. Ed.** .. .. *Index of Differential Diagnosis* .. 4th Ed. 1928
**Garré, C., und Borchard, A.** *Lehrbuch der Chirurgie* .. .. 6te Aufl. (6) 1928
**Gordon, R. G.** .. .. *Autolycus* .. .. .. .. .. .. .. .. .. .. .. .. 1928
**Gouda, S.** .. .. .. *La synthaline dans le traitement du diabete* .. (3) 1927
**Gould, A. P.** .. .. *Elements of Surgical Diagnosis* .. 7th Ed. 1928
**Green-Armytage, V. B.** *Tropical Midwifery* .. .. .. .. .. .. .. .. (4) 1928
**Green-Armytage, V. B.** *Tropical Gynæcology* .. .. .. .. .. .. .. 1928
**Hale-White, W.** .. .. *Materia Medica* .. .. .. .. .. .. 19th Ed. (8) 1927
**Hawthorne, C. O.** .. *Short Essays on Medical Topics* .. .. .. .. .. 1928
**Howard, R.** .. .. *Practice of Surgery* .. .. .. .. .. 3rd Ed. (8) 1924
*Index Catalogue of the Surgeon-General's Library* 3rd Series, Vol. 7 (13) 1928
*Index of Symptomatology*, ed. Tidy .. .. .. .. .. .. .. .. .. .. .. .. 1929
**Kirschner, M., und Schubert, A.** *Operationslehre* .. .. .. Bd. I. (6) 1927
**Köhler, A.** .. .. .. *Röntgenology.* Transl. Turnbull .. .. .. (6) 1928
**Lamb, W.** .. .. .. *Diseases of the Throat, Nose and Ear* 5th Ed. (8) 1927
**Leriche, R., and Policard, A.** *The Normal and Pathological Physiology of Bone.* Transl. Moore and Key .. (6) 1928
**MacCallum, W. G.** .. *Textbook of Pathology* .. .. .. 4th Ed. (8) 1928
**Maclean, H.** .. .. *Modern Methods in the Diagnosis and Treatment of Renal Disease* .. .. .. .. .. 3rd Ed. (8) 1927
**Montague, J. F.** .. *Taking the Doctor's Pulse* .. .. .. .. .. .. 1928
**Moore, I.** .. .. .. *The Tonsils and Adenoids* .. .. .. .. .. .. 1928
**Moxon, H. W.** .. .. *A Patient's Manual of Diabetes* .. .. .. .. .. 1929

| | | | | |
|---|---|---|---|---|
| Mozer J. J. | *Les ostéoscléroses diffuses les anémies ostéo-sclérotiques* | | (3) | 1927 |
| Muskens, L. J. J. | *Epilepsy* | | (8) | 1928 |
| Oettingen, W. von | *Chirurgie* | | (6) | 1928 |
| Pachon, V. et Fabre, R. | *Le cardiogramme* | | (1) | 1929 |
| Petrovitch, M. | *Un cas d'anémie aplastique* | | (3) | 1928 |
| Piney, A. | *Diseases of the Blood* | | (8) | 1928 |
| Piney, A. | *Recent Advances in Hæmatology* | 2nd Ed. | (8) | 1928 |
| Pritchard, E. | *Physiological Feeding of Infants* | 4th Ed. | (8) | 1923 |
| Quervain, F. de | *Clinical Surgical Diagnosis* | 4th Ed. | (8) | 1926 |
| Robertson, W., and Porter, C. | *Sanitary Law and Practice* | 6th Ed. | (8) | 1928 |
| Rose and Carless | *Manual of Surgery* | 12th Ed. | (6) | 1927 |
| Rosenau, M. J. | *Preventive Medicine and Hygiene* | 5th Ed. | (8) | 1927 |
| Runting, E. G. V. | *Practical Chiropody* | 3rd Ed. | | 1928 |
| Sayegh, A. | *Un cas d'endocardite septique* | | (3) | 1927 |
| Schönbauer, L. | *Konservative Frakturenbehandlung* | | (6) | 1928 |
| Shattock, C. E. | *Handbook of Surgical Diagnosis* | | (6) | 1929 |
| Souttar, H. S. | *Art of Surgery* | | | 1929 |
| Taylor, A. S. | *Principles and Practice of Medical Jurisprudence* 2 vols. | 8th Ed. | (8) | 1928 |
| Tomes, C. S. | *Manual of Dental Anatomy* | 8th Ed. | (8) | 1923 |
| Ward, E. | *Medical Adventure* | | | 1929 |
| Widdowson, T. W. | *Notes on Dental Anatomy and Physiology and Dental Histology* | 5th Ed. | (8) | 1928 |
| Williams, J. W. | *Obstetrics* | 5th Ed. | (8) | 1927 |
| Woollard, H. | *Recent Advances in Anatomy* | | (8) | 1927 |
| Young, H. H. | *Practice of Urology* | 2 vols. | (8) | 1927 |
| Ziegner, H. | *Vademekum der speziellen Chirurgie und Orthopadie* | 9te Aufl. | (6) | 1928 |

TRANSACTIONS, REPORTS, JOURNALS, ETC.

| | | |
|---|---|---|
| Cancer Review. Vol. I. and in continuation | (8) | 1929 |
| English Catalogue of Books, 1928 | | 1928 |
| Medical Society of London Transactions. Vol. LI. | | 1928 |
| Methods and Problems of Medical Education. 11th Series | (11) | 1928 |
| Minutes of the G.M.C. Vol. LXV and Index 1903–1928 | (10) | 1929 |
| Progressive Medicine. December, 1928 | | |
| Smithsonian Institution. Annual Report, 1927 | | 1928 |
| Surgical Clinics of North America. December, 1928 | | |
| Transactions of the Association of American Physicians, Vol. XLIII. | | 1928 |

### Local Medical Notes.

APPOINTMENTS.

Colonel F. P. MACKIE, I.M.S., V.H.S., O.B.E., M.D., F.R.C.S., F.R.C.P., M.Sc., D.P.H., to be Honorary Surgeon to His Majesty the King.

**Royal College of Surgeons.**—Professor E. W. Hey Groves has been appointed to deliver the Hunterian Oration for 1930.

EXAMINATION RESULTS.

**University of Bristol.**—Students of the University have recently passed the following examinations :—

M.B., CH.B (*Part I.*)—*Second Examination. Pass :* S. B. Adams, A. J. Board, J. F. H. Eagles, Violet Fry, Margaret A. W, Hawkes, Winifrede M. Hill, A. V. House, F. J. W. Lewis. H. E. Pearce, Frances E. Powell, O. E. L. Sampson. *In Organic Chemistry* (*Completing Examination*) *:* H. James. *In Elementary Anatomy only :* Mary G. Thomas, S. P. N. Williams.

M.B., CH.B. (*Part II.*).—*Second Examination. Pass :* G. T. Bevir, R. N. O. Burns, R. G. C. G. Carlson, A. M. Davies, G. M. Evans, A. L. Eyre-Brook (with distinction in Anatomy), H. F. M. Finzel (with distinction in Anatomy), F. E. Fletcher, J. D. Hughes, J. J. Kempton R. L. Marks, J. Ridgway, Frances N. Salisbury, O. E. L. Sampson, J. P. P. Stock (with distinction in Anatomy).

L.D.S.—*First Professional Examination. Pass : In Anatomy and Physiology* (*Completing Examination*), W. V. Whiteford. *In Dental Anatomy only :* R. E. W. Smith.

CONJOINT BOARD.—*In Physics :* A. V. House, S. P. N. Williams. *In Pharmacology and Materia Medica :* W. Woolley. *In Medicine :* W. H. B. Spoor, T. Evans, G. J. S. West, R. G. Mayer. *In Surgery :* T. Evans, April D. James, J. D. Williams, N. D. Gerrish. *In Midwifery :* T. Evans, G. J. S. West, R. G. Mayer, C. E. Brierley, April D. James.

M.R.C.S., L.R.C.P.—T. Evans, J. D. Williams.

L.D.S., R.C.S.—*First Professional Examination* (*Part I.*) *:* I. M. Ayliffe, G. E. Chadd, G. H. Dakers, C. A. G. Evans, K. V. M. Taylor-Milton. *Part II. :* Q. A. Davies, J. K. Noot. *Part III.* (*a*) *:* Beryl M. Davies, W. A. Duly. *Part III.* (*b*) *:* G. H. Dakers, E. S. Edwards, W. U. Harwood, A. M. Watson, J. H. White.

L.D.S., R.C.S.—*Final* (*Part I.*) *:* F. G. Lilley, J. P. Price, J. D. Sellars. *Part II. :* C. Haskins.

L.M.S.S.A.—T. A. Barnabas passed in Surgery, completing examination. L. P. Gregory passed in Medicine, completing examination.

### Bristol Medico-Chirurgical Society.

### President.

H. L. ORMEROD, M.D., B.Ch., R.U.I.

### President-Elect.

C. FERRIER WALTERS, F.R.C.S.

### Committee.

*Ex-Officio* { J. A. NIXON, *C.M.G.*, B.A., M.D. Cantab. (Editor of *Journal*).

W. A. SMITH, M.A., M.B. Oxon. (Hon. Librarian).

*Elected.*

A. L. FLEMMING, M.B., Ch.B. Brist. }
T. CARWARDINE, M.S. Lond. } Ex-Presidents.
W. K. WILLS, *O.B.E.*, M.A., M.B., B.Ch. Cantab. }
D. C. RAYNER, F.R.C.S.
W. VINCENT WOOD, *M.C.*, B.A. Cantab.
DUNCAN WOOD, F.R.C.S.
R. S. S. STATHAM, *O.B.E.*, M.D., M.Ch. Brist.
H. CHITTY, M.S., F.R.C.S.
C. H. TERRY, M.A., M.B., B.Ch. Oxon.

### Honorary Secretary.

W. A. JACKMAN, M.B., F.R.C.S. Ed.

### Honorary Treasurer.

A. E. ILES, *O.B.E.*, M.B., B.S., F.R.C.S.

### University Library Sub-Committee.

W. A. SMITH, M.B., M.A. Oxon. G. PARKER, M.D., M.A. Cantab.
A. R. SHORT, M.D.

Medical Practitioners wishing to join the Society are requested to communicate with the Hon. Secretary, Mr. W. A. JACKMAN, 15 Mortimer Road, Clifton. The Annual Subscription is £1 11s. 6d., payable to Hon. Treasurer.

---

*Extracts from Journal By-laws.*

4.—The *Journal* shall be delivered free to Subscribers and also to Members of the Society whose subscriptions are not in arrear.

6.—The Annual Subscription to the *Journal* is 10/6, payable to Hon. Treasurer.

---

Communications intended for insertion in the *Journal* should be sent to the Editor, Dr J. A. NIXON, 7 Lansdown Place, Clifton, Bristol.

Books for Review and Exchange Journals should be sent to the Assistant-Editor, *Bristol Medico-Chirurgical Journal*, Medical Library, The University, Bristol.

Communications referring to the delivery of the *Journal* should be sent to the Editorial Secretary, A. L. Flemming, 48 Pembroke Road, Clifton, Bristol.

Cases for Binding Vols. I—XLV are now ready, and may be obtained, price 1/9 each (postage extra), upon application to J. W. ARROWSMITH LTD., Quay Street, Bristol; or the *Journal* will be bound, including Case, for 7/- per volume.

### Bristol Medico-Chirurgical Society.

### PAST PRESIDENTS.

1874—75. FREDERICK BRITTAN, M.D., B.A. Dub.
1875—76. ROBERT WILLIAM COE.
1876—77. SAMUEL MARTYN, M.D. Ed.
1877—78. AUGUSTIN PRICHARD, M.D. Berlin.
1878—79. HENRY EDWARD FRIPP, M.D. Würzburg.
1879—80. WILLIAM MICHELL CLARKE.
1880—81. JOSEPH GRIFFITHS SWAYNE, M.D. Lond.
1881—82. EDWARD LONG FOX, M.D. Oxon.
1882—83. JAMES GEORGE DAVEY, M.D. St. And.
1883—84. WILLIAM JOHNSTONE FYFFE, M.D., B.A. Dub.
1884—85. GEORGE FORSTER BURDER, M.D. Aberd.
1885—86. WILLIAM HENRY SPENCER, M.D., M.A. Cantab.
1886—87. FRANCIS POOLE LANSDOWN.
1887—88. LEMUEL MATTHEWS GRIFFITHS.
1888—89. ROBERT SHINGLETON SMITH, M.D., B.Sc. Lond.
1889—90. NELSON CONGREVE DOBSON, Ch.M. Brist.
1890—91. SAMUEL HENRY SWAYNE.
1891—92. FRANCIS RICHARDSON CROSS, M.B. Lond., LL.D. Brist.
1892—93. EDWARD MARKHAM SKERRITT, M.D., B.S., B.A. Lond.
1893—94. JAMES GREIG SMITH, M.B., C.M., M.A. Aberd., F.R.S.E.
1894—95. ALFRED JAMES HARRISON, M.B. Lond.
1895—96. ARTHUR WILLIAM PRICHARD.
1896—97. ALFRED EDWARD AUST LAWRENCE, M.D., C.M. Aberd.
1897—98. JOHN EDWARD SHAW, M.B., C.M. Ed.
1898—99. ROBERT ROXBURGH, M.B. Ed.
1899—00. WILLIAM HENRY HARSANT.
1900—01. DAVID SAMUEL DAVIES, M.D. Lond.
1901—02. BARCLAY JOSIAH BARON, M.B., C.M. Ed.
1902—03. GEORGE MUNRO SMITH, M.D. Brist.
1903—04. JAMES PAUL BUSH, C.M.G., C.B.E., Ch.M. Brist.
1904—05. REGINALD EAGER, M.D. Lond.
1905—06. JOHN DACRE.
1906—07. JAMES TAYLOR.
1907—08. HENRY WALDO, M.D., C.M. Aberd.
1908—09. JOHN MICHELL CLARKE, M.D., M.A. Cantab.
1909—10. JAMES SWAIN, C.B., C.B.E., M.D., M.S. Lond.
1910—11. BERTRAM MITFORD HERON ROGERS, M.D., B.Ch., B.A. Oxon.
1911—12. CHARLES ALEXANDER MORTON.
1912—13. WALTER CARLESS SWAYNE, M.D., B.S. Lond. and Brist.
1913—14. PATRICK WATSON-WILLIAMS, M.D. Lond.
1914—15. WILLIAM HARRY CHRISTOPHER NEWNHAM, M.A., M.B. Cantab.
1915—16 GEORGE PARKER, M.A., M.D. Cantab.
1916—17 FRANCIS HENRY EDGEWORTH, M.A., M.D. Cantab., D.Sc. Lond.
1917—18 FREDERICK LACE.
1918—19 ROBERT GUTHRIE POOLE LANSDOWN, M.D., B.S. Durh.
1919—20 I. WALKER HALL, M.D., Ch.B. Vict.
1920—21 LEOPOLD ERNEST VALENTINE EVERY-CLAYTON, M.D. Lond.
1921—22 CYRIL HUTCHINSON WALKER, M.B., B.A. Cantab.
1922—23 JOHN ALEXANDER NIXON, C.M.G., B.A., M.D. Cantab.
1923—24 JOHN LACY FIRTH, M.D., M.S. Lond.
1924—25 JOHN ODERY SYMES, M.D. Lond.
1925—26 THOMAS CARWARDINE, M.S. Lond.
1926—27 ARTHUR LAUNCELOT FLEMMING, M.B., Ch.B. Brist.
1927—28 W. KENNETH WILLS, M.A., M.B., B.Ch. Cantab.

1929.

### LIST OF SUBSCRIBERS

TO THE

### Bristol Medico-Chirurgical Journal.

### HONORARY MEMBERS OF THE SOCIETY.

| | |
|---|---|
| SWAIN, JAMES, *C.B.*, *C.B.E.*, M.D., M.S. .. .. .. .. .. | Wyndham House, Easton-in-Gordano. |
| WATSON-WILLIAMS, P., M.D. .. | 2 Rodney Place, Clifton. |
| PAUL BUSH, J., *C.M.G.*, Ch.M. | The Clifton Club. |

### SUBSCRIBERS.

*Those marked * are Members of the Society.*

| | |
|---|---|
| *ADAMS, A. W., M.S. Lond. ... ... ... ... ... | 9 Mortimer Road, Clifton. |
| ADYE, W. J. H. ... ... ... ... ... ... ... | St. Margaret's, Bradford-on-Avon. |
| *ALEXANDER, A., M.A. ... ... ... ... ... ... | 23 Manilla Road, Clifton. |
| *ALEXANDER, D. A., M.B., Ch.B. Vict. ... ... | 112 Pembroke Road, Clifton, Bristol. |
| ALEXANDER, G. L., B.A. Cantab. ... ... ... | Principal Medical Officer, Freetown, Sierra Leone. |
| *ALFORD, A. F., M.B., Ch.B. Bristol ... ... ... | "Drofla," Kellaway Avenue, Westbury Park, Bristol. |
| ALFORD, H. J., M.D. ... ... ... ... ... ... | 4 Park Terrace, Taunton. |
| *ASKINS, R. A., M.D., B.Ch., B.A.O. Dub. ... ... | Guildhall, Bristol. |
| *AUBREY, T., M.B. Lond. ... ... ... ... ... | Bitton, near Bristol. |
| *BAKER, LILY A., M.D., B.Ch., B.A.O., R.U.I. ... | Vyvyan House, Clifton. |
| *BARBER, M. C., M.B., Ch.B. Brist. ... ... ... | Ingledene, High Street, Staple Hill, Bristol. |
| *BARKER, G. H., M.D., B.Sc. Lond. ... ... ... | 124 Redland Road, Bristol. |
| *BARTHOLOMEW, E. U. ... ... ... ... ... ... ... | 12 Lansdown Place, Clifton. |
| *BATT, J. C., M.B., Ch.B. Bristol ... ... ... | Bristol General Hospital. |
| BEATSON, D. H., M.B., Ch.B. ... ... ... ... | 8 Richmond Park Road, Clifton. |
| *BENSON, ELIZABETH, M.B., Ch.B. Brist. ... ... | 8 Theresa Place, Bristol Road, Gloucester. |
| *BERGIN, F. G. ... ... ... ... ... ... ... | 31 Oakfield Road, Clifton. |
| BERNARD, C. ... ... ... ... ... ... ... ... | 564 Fishponds Road, Fishponds, Bristol. |
| *BERRILL, T. H., M.B., Ch.B. Bristol ... ... | Bristol Royal Infirmary. |
| BERRY, F. C., M.D., B.Ch., B.A. Dub. ... ... | Locarno, Burnham, Somerset. |
| *BIRRELL, J. A., M.D., M.S. Lond. ... ... ... | 2 Dundonald Road, Redland, Bristol. |
| *BLACHFORD, J. V., *C.B.E.*, M.D. Durh. ... ... | Milverton House, Long Ashton. |
| BLACKETT, J. F., M.D., B.S. Lond. ... ... ... | Hamsterley, Bloomfield Park, Bath. |
| *BLAGG, ARTHUR F., M.D. Durh. ... ... ... ... | 6 Upper Belgrave Road, Clifton. |
| *BLATHWAYT, A. de V. ... ... ... ... ... ... | 6 Brock Street, Bath. |
| BLETCHLY, G. PLAYNE, M.B. Lond. ... ... ... | Hazelwood, Nailsworth, Glos. |
| *BODMAN, A. P., M.B., Ch.B. ... ... ... ... | 11 Hurle Crescent, Clifton, Bristol. |
| *BODMAN, C. O., M.D. Durh. ... ... ... ... | 17 Upper Belgrave Road, Clifton. |
| *BODMAN, F. H., M.B., Ch.B. Brist. ... ... ... | 170 Cheltenham Road, Bristol. |

| | |
|---|---|
| *Bodman, J. Hervey, M.D. Lond. | 11 Hurle Crescent, Clifton, Bristol. |
| *Bradbeer, E. G., M.B., Ch.B. Brist. | 32 Linden Road, Westbury Park, Bristol. |
| *Bradley, W. H., B.M., B.Ch. Oxon. | St. Wulstan's, Stratton-on-Fosse, Bath. |
| *Branthwaite, C.B., M.D., D.P.H. | Stoke Park, Stapleton. |
| *Bray, G., Lt.-Col., *D.S.O.* | 46 Henleaze Avenue, Westbury-on-Trym. |
| *Brentnall, T. C. | Bellfield, Brightlingsea, Essex. |
| *Boulton, B. J., M.B., Ch.B. Bristol | Bristol General Hospital. |
| *Bristowe, H. C., M.D. Lond. | Wrington, Somerset. |
| *Brittain, B. A., M.B., Ch.B. Dublin | Bristol Royal Infirmary. |
| *Britton, R. B., M.B., B.S. Lond. | Troy House, Kingswood, Bristol. |
| *Buckmaster, G. A., M.A., M.D. Oxon. | University of Bristol. |
| *Burdon-Cooper, J., M.D., B.Sc. Durh. | 12 The Circus, Bath. |
| *Burges, R. | Druid's Mead, Stoke Bishop, Bristol. |
| *Burgess, N. | Hereford House, Clifton. |
| *Burnett, R. H., M.B., B.S. Durh. | 479 Bath Road, Brislington, Bristol. |
| *Bush, G. B., M.B., Ch.B. Brist. | 5 Lansdown Place, Clifton. |
| | |
| *Cairns, F. J. | Devon House, Whitehall Road, Bristol. |
| *Carey, R. S., *O.B.E.*, B.A. Cantab. | 502 Bath Road, Brislington, Bristol. |
| *Carleton, H. H., M.A., M.D., B.Ch. Oxon | 1 Lansdown Road, Clifton. |
| *Carling, A., M.A., M.B., B.C. Cantab. | 12 Great George Street, Bristol. |
| *Carter, T. M., *O.B.E.*, M.D. | 19 Westfield Park, Redland, Bristol. |
| *Carwardine, Thomas, M.B., M.S. Lond. | 16 Victoria Square, Clifton. |
| *Cates, H. J., M.D. Lond. | Northwoods House, Winterbourne, Bristol. |
| *Cave, Edward J., M.D. Lond. | 16 The Circus, Bath. |
| *Chambers, E. R. | 9 Clifton Park, Clifton. |
| *Charles, J. R., M.A., M.D., B.C. Cantab. | 1 Victoria Square, Clifton. |
| *Chesterman, J. T., M.D., B.S. | Bristol Royal Infirmary. |
| *Chitty, H., M.S. Lond. | 46 Pembroke Road, Clifton. |
| *Christofferson, P. E., M.B., Ch.B. Brist. | 8 Lansdown Place, Clifton. |
| *Claremont, L. E. | 5 Rodney Place, Clifton. |
| *Clarke, R. C., *O.B.E.*, M.B., Ch.B. Brist. | 29 Victoria Square, Clifton. |
| *Coates, Vincent M., *M.C.*, M.A., M.D., B.C. Cantab. | 10 The Circus, Bath. |
| Connellan, P. S. | 12 Bloomsbury Street, London, W.1. |
| *Cookson, R. G., L.R.C.P., L.R.C.S. Ireland | Clifton Dispensary, Bristol. |
| *Coombs, Carey, M.D. Lond. | 3 Pembroke Road, Clifton. |
| *Cooper, William Russell | 13 Westfield Park, Redland, Bristol. |
| *Corfield, C., M.D. Durh. | 5 Kensington Place, Clifton. |
| *Cory, R. A. S. | Bristol Royal Infirmary. |
| *Cossham, W. L., M.B., Ch.B. Bristol | 32 Somerville Road, St. Andrew's Park, Bristol. |
| *Coxon, Beatrice | 16 Richmond Hill, Clifton. |
| Cridland, A. B. | Salisbury House, Chapel Ash, Wolverhampton. |
| *Cross, R. F., M.B. Lond., LL.D. Brist. | Worcester House, Clifton. |
| *Crossman, F. W., M.B. Durh. | White's Hill, Hambrook, near Bristol. |
| | |
| *Dacre, John | 14 Eaton Crescent, Clifton. |
| *Davies, E. D. D. | Standish House, Stonehouse, Glos. |
| Devine, H., *O.B.E.*, M.D. Lond. | Holloway Sanatorium, Virginia Water. |
| *Dawe, J. H., M.B. Ed. | Lynwood House, Bickford Road, Bath. |
| *Dix, C. | 11 Victoria Square, Clifton. |
| *Dixon, Helen M., M.B., B.Ch. Brist. | 10 Whatley Road, Clifton. |
| *Dixon, T. B. | 11 Pembroke Road, Clifton, Bristol. |
| *Drapes, T. L., M.B., B.C., B.A. Cantab. | St. Maur, Beaufort Square, Chepstow. |
| *Duerden, J. R., M.B., Ch.B. Brist. | 149 Bell Hill Road, St. George, Bristol. |
| *Dutton, Hilda R. | 215 Stapleton Road, Bristol. |
| *Dykes, A. L., M.D. Lond. | 12 St. Julian Road, Bristol. |

| | |
|---|---|
| *Egdeworth, F. H., M.D., B.C., M.A. Cantab., D.Sc. Lond. | 13 Richmond Hill, Clifton. |
| *Eglinton, J. H. C. | Street, Somerset. |
| *Elkington, G. W., M.B., B.S. Lond. | Bruton, Somerset. |
| Elliot Bros, Ltd. | *Australian Pharmaceutical Notes & News*, O'Connell Street, Sydney, N.S.W., c/o Grimwade, Ridley & Co., 124 Minories, E.C. |
| Elliott, F. Percy, M.B., C.M. Aberd. | 113 Grove Rd., Walthamstow, London, E.17. |
| *Elliott, W. H. A., M.B., B.S. Lond. | 116 Pembroke Road, Clifton. |
| *Every-Clayton, L. E. V., M.D. Lond. | The Quarry, Mullion, Cornwall. |
| *Faill, C. J. C. | 19 Portland Square, Bristol. |
| *Fallon, Col. J. | 35 Cavendish Road, Henleaze, Bristol. |
| *Faraker, E. C. | Keinton Mandeville, Somerset. |
| Farnfield, W. W. | Clive Vale, Gillingham, Dorset. |
| *Fawn, G. F. | 6 Oakfield Road, Clifton. |
| *Fells, A., M.B., C.M. Ed. | 6 Elton Road, Clifton. |
| *Fells, G. R., M.B., Ch.B. Bristol | Bristol General Hospital. |
| *Fells, R. R., M.B., B.S. Lond. | 12*d* Cotham Road, Bristol. |
| *Fisher, A. C. | Bristol Royal Infirmary. |
| *Firth, J. L., M.D., M.S. Lond. | 8 Victoria Square, Clifton. |
| *Flemming, A. L., M.B., Ch.B. Brist. | 48 Pembroke Road, Clifton. |
| *Flemming, C. E. S. | Manvers House, Bradford-on-Avon. |
| *Forrester-Brown, Maud, M.D., M.S. | 22 Coombe Park, Bath. |
| *Foss, E. V., M.D., Ch.B. Brist. | Summer Hill House, St. George, Bristol. |
| *Fox, F. E., B.A. Camb. | Brislington House. |
| *Fraser, A. D., M.A., M.B., Ch.B. Glasg. | 4 Alexandra Road, Clifton. |
| *Fuller, H. L. | 9 Gay Street, Bath. |
| *Garden, R. R., M.A., M.B., B.Ch. | 9 Harley Place, Clifton. |
| Garland, E. B., M.B., C.M. Ed. | 114*a* Hampton Road, Redland, Bristol. |
| *Gee, C. A. H., M.B., Ch.B. Brist. | Portbury, Nr. Bristol. |
| *Gibbs, A. N. Godby | 30 Whiteladies Road, Clifton. |
| *Glenny, E. T., M.B., B.S. Lond. | 102 Pembroke Road, Clifton. |
| *Gordon, R. G., M.D., B.Sc. Ed. | 9 The Circus, Bath. |
| *Gorham, A. P., M.B., Ch.B. | 53 Westbury Road, Westbury-on-Trym. |
| *Gornall, W. A., M.B., Ch.B. Brist. | 23 Windsor Road, St. Andrew's Park, Bristol. |
| *Green, T. A., *D.S.O.*, M.D., C.M. Ed. | 33*a* Whiteladies Road, Clifton. |
| *Greenlaw, Madge E., M.B., Ch.B. Brist. | 194 Redland Road, Bristol. |
| Griffiths, Cornelius A. | 35 Newport Road, Cardiff. |
| *Griffiths, J. S. | Redland Park House, Bristol. |
| *Griffiths, S. J. H., M.B., Ch.B. Brist. | Bristol General Hospital. |
| *Groves, E. W. Hey, M.D., M.S. Lond. | 24 Victoria Square, Clifton. |
| *Hadfield, G., M.D. Lond. | Royal Free Hospital, London. |
| *Hailes, Clements, D. G., M.D., C.M. Ed. | 2 Richmond Park Road, Clifton. |
| *Hall, E. George, M.B. Lond. | 42 Apsley Road, Clifton, Bristol. |
| *Hall, I. Walker, M.D., Ch.B. Vict. | Shortwood Lodge, Pucklechurch, Glos. |
| *Ham, C. I., M.B., Ch.B. Brist. | Bristol Royal Infirmary. |
| *Harris, H. Elwin, B.A., M.B. Cantab. | 13 Lansdown Place, Clifton |
| *Harris, H. E., M.A., M.B., B.Ch. Cantab. | 13 Lansdown Place, Clifton. |
| *Harsant, W. H. | Tower House, Clifton Down Road, Clifton. |
| *Hector, F., M.D. Lond. | Chelsea Hospital for Women, Arthur Street, Chelsea, S.W.3. |
| *Herapath, C. E. K., *M.C.*, M.D. Lond. | Ormlie, Clifton. |
| *Hooker, J. A., M.B., Ch.B. | 16 St. Edyth's Road, Sea Mills. |
| Hosken | Uley, Glos. |

Hospital, Bristol General (*Secretary*, T. W. Gregg).

*Houghton, L. M., M.B., B.S. Lond. ... ... 74 Church Road, Redfield, Bristol.
*Hughes, T. I. ... ... ... ... ... ... ... ... Bristol General Hospital.
*Hughes F. W. Terrell, M.B., Ch.B. Brist. ... Chew Magna, Somerset.
Hughes, D., M.B., B.Ch., B.A.O., N.U.I. ... 12*d* Cotham Road, Bristol.
*Hunter, F. S. ... ... ... ... ... ... ... ... c/o Messrs. J. Wright & Sons Ltd., Publishers, Bristol.

*Iles, A. E., *O.B.E.*, M.B., B.S. Lond. ... ... 17 Victoria Square, Clifton.
Infirmary, Bristol Royal (*Secretary*, E. C. Smith).
Ingle, C. Durban ... ... ... ... ... ... ... Somerton, Somerset.

*Jackman, W. A., M.B., Ch.B. Brist. ... ... ... 15 Mortimer Road, Clifton.
*James, J. A., M.D. Lond., F.R.C.S. ... ... ... 5 Rodney Place, Clifton.
*Jenkins, R. D. ... ... ... ... ... ... ... ... General Hospital, Bristol.
*Johnson, R. G., M.B. Lond. ... ... ... ... ... 119 Redland Road, Bristol.
*Joll, C. A., M.B., M.S. Lond. ... ... ... ... 64 Harley Street, London.

*Kemm, N. F., M.B., B.S. Lond. ... ... ... 188 Redland Road.
*Kennedy, W. P., M.D. Dub. ... ... ... ... ... 3 Gay Street, Bath.
*Kerfoot, Stanley J. ... ... ... ... ... ... ... 194 Redland Road, Bristol.
*Kingston, S. H., M.B., Ch.B. Brist. ... ... 3 Whiteladies Road, Clifton.
*Kyle, H. G., M.A., M.D., B.Ch. Oxon. ... ... 31 Westbury Road, Bristol.

*Lace, F. ... ... ... ... ... ... ... ... ... ... ... 5 Gay Street, Bath.
*Lees, E. Leonard, M.D., C.M. Ed. ... ... ... 22 Richmond Hill, Clifton.
*Legat, R. Eddowes, M.B., C.M. Ed. ... ... Murray House, Staple Hill, Bristol.
Leigh, W. W. ... ... ... ... ... ... ... ... Llansamnor House, Cowbridge, Glam.
*Leonard, R. C., M.D. Durh. ... ... ... ... Bower Ashton, Bristol.
*Lester, Muriel A., M.B., B.S. ... ... ... 110 Avon Vale Road, Redfield, Bristol.
*Levis, J. S., *M.C.*, M.B., B.Ch., N.U.I. ... ... 20 Bay Street, Bath.
*Linton, Marion S., M.B. Lond. ... ... ... 21 Oakfield Road, Clifton.
*Lister, A. E. J., M.B., B.S. Lond., F.R.C.S. ... 86 Pembroke Road, Clifton.
*Lister, L. Margaret ... ... ... ... ... ... ... 12 All Saints Road, Clifton.
*Logan, H. B. ... ... ... ... ... ... ... ... ... Elmwood, Aberdeen Road, Clifton.
*Lucas, J. E. ... ... ... ... ... ... ... ... ... 48 Coronation Road, Bristol.
*Lucas, J. J. S., M.D. Lond. ... ... ... ... 186 Wells Road, Totterdown, Bristol.
*Lysaght, A. C. ... ... ... ... ... ... ... ... 8 Clifton Hill, Bristol.

*Macfie, J. D., M.B., Ch.B. ... ... ... ... Winsley Sanatorium, near Bath.
Mackinnon, Charles, M.B., C.M., M.A. Glasg. Davaar, Cirencester.
Maclean, Sir Ewen J., M.D., C.M. Ed. ... ... 12 Park Place, Cardiff.
*MacLeod, Donald, M.B., C.M. Ed. ... ... ... 84 Redland Road, Redland, Bristol.
*MacLeod, G., *M.C.*, M.D., Ch.B. ... ... ... "Wargrave," Clevedon.
*MacWatters, J. C., *M.B.E.* ... ... ... ... Almondsbury, near Bristol.
Marle, S. ... ... ... ... ... ... ... ... ... ... Newbridge Road, Bath.
Marshall, Arthur L., M.B., B.A. Cantab. ... Raveningham, Norwich.
Mather, J. S., M.B., C.M. Aberd. ... ... ... Lawrence Hill House, Bristol.
*Mathew, W. E., M.D., C.M. Toronto ... ... 85 Coldharbour Road, Redland.
*Mayes, F. J. A. ... ... ... ... ... ... ... ... 79 Pembroke Road, Clifton.
*McKenzie, W. G., *M.C.* ... ... ... ... ... ... 101 Coronation Road, Bristol.
*McLannahan, J. G. ... ... ... ... ... ... ... The Mount, Stonehouse.
Miall, C. L'O ... ... ... ... ... ... ... ... Elm Hayes, Paulton, near Bristol.
*Middleton-West, S. H., *M.C.*, M.B., Ch.B. Vict. 28 Percival Road, Clifton.
*Milling, T., M.B., Ch.B. Belfast ... ... ... 1 Vicarage Road, Southville.
*Milner, A., M.B., B.Ch. Dublin ... ... ... 11 Freemantle Square, Kingsdown, Bristol.
*Moore, C. A., M.B., M.S. Lond. ... ... ... 56 St. Paul's Road, Clifton.
*Moore, L. A. ... ... ... ... ... ... ... ... ... Hillsborough, Cotham Park, Bristol.
*Morris, A. G. ... ... ... ... ... ... ... ... 18 Westbury Park, Westbury-on-Trym.

| | |
|---|---|
| *Morris, L. N., M.B., Ch.B. Brist. | 24 All Hallows Road, Upper Easton, Bristol. |
| *Morton, C. A., *O.B.E.* | 14 Vyvyan Terrace, Clifton. |
| *Mumford, W. G., M.B. Lond. | 18 The Circus, Bath. |
| *Mundy, G. S., M.B., B.Ch. Brist. | Homewood, Coombe Dingle, Bristol. |
| *Murphy, F. D., M.B., Ch.B., B.Sc. Cork | Southmead Hospital, Bristol. |
| *Myles, G. T. | 7 Apsley Road, Clifton. |
| | |
| *Neild, Newman, M.B., B.Ch. Vict. | 9 Richmond Hill, Clifton. |
| *Newnham, W. H. C., M.B., M.A. Cantab. | 3 Lansdown Place, Victoria Square, Clifton. |
| *Nichols. F. C., *M.C.*, M.B., Ch.B. Brist. | 38 Whiteladies Road, Clifton. |
| *Nixon, J. A., *C.M.G.*, M.D. Cantab. | 7 Lansdown Place, Clifton. |
| *Noble, J. Innes, M.B., Ch.B. | 102 Pembroke Road, Clifton. |
| *Nott, Winifred, M.B., Ch.B. Brist. | "Fassiefern," Upper Cranbrook Road, Redland, Bristol. |
| | |
| *Ormerod, H. Lawrence, M.D., B.Ch. R.U.I. | 2 Henleaze Road, Westbury-on-Trym, Bristol. |
| Orr-Ewing, H. J., *M.C.*, M.D. Lond. | English Mission Hospital, Jerusalem. |
| | |
| *Packer, M. E. J., M.B., Ch.B. Brist. | 90 Filton Road, Filton, Bristol. |
| *Parker, George, M.D., M.A. Cantab. | 14 Pembroke Road, Clifton. |
| *Parkes, J. A., M.B., Ch.B. Liverpool | 8 South Road, Redland, Bristol. |
| Parkinson, Lt.-Col. G. S., *D.S.O.*, R.A.M.C. | R.A.M.C. Mess, Grosvenor Place, London, S.W.1. |
| *Parry, R. H., M.B., B.S. Lond. | Newby, Holmes Grove Road, Henleaze. |
| Parsons, Sir J. H., M.B., B.S., B.Sc. Lond. | 54 Queen Anne Street, London, W. |
| *Pearce, J. L., M.B., Ch.B. Edin. | Hambrook Court, near Bristol. |
| *Perry Bruce, M.B., Ch.B. | The General Hospital, Bristol. |
| *Phillips, P., M.B., Ch.B. Brist. | Southmead Hospital, Bristol. |
| *Pinkerton, J. M., F.R.C.S. | 5 The Circus, Bath. |
| *Pollard, J., M.B., B.Ch., B.A.O. | 88 Coronation Road, Bristol. |
| *Powell, J. J., M.D. Lond. | 2 Gloucester Road, Bishopston, Bristol. |
| *Price, H. H., M.B., C.M. Ed. | 17 Oakfield Road, Clifton. |
| Prowse, D. C., M.B., Ch.B. Brist. | Wigmore House, Thornbury, nr. Bristol. |
| *Pyke, H. D., M.B., Ch.B. Brist. | 19 Mount Gold Road, Plymouth. |
| | |
| *Rayner, D. C. | 9 Lansdown Place, Clifton, Bristol. |
| *Renton, R. A. S., *M.C.*, M.D. Durh. | Emsworth House, Clevedon. |
| Robertson, D., M.B., Ch.B. Ed. | 205 Wells Road, Knowle, Bristol. |
| *Rogers, B. M. H., M.D., B.Ch., B.A. Oxon. | 14 Mortimer Road, Clifton. |
| *Rogers, Herbert, M.B., Ch.B. | 11 Clifton Hill, Bristol. |
| *Rolfe, R., B.A., M.B., B.C. Cantab. | Park House, St. Andrews Road, Avonmouth. |
| *Ross, C., M.D. | Bristol Royal Infirmary. |
| *Rowley, A. T. | Wrington. |
| *Rutherford, J. M., M.B., C.M. Ed. | Brislington House, near Bristol. |
| | |
| *Salisbury, Walter, M.D., M.S. Lond. | Woodlands, 20 Billing Road, Northampton. |
| Savage, W. G., M.D., B.Sc. Lond. | "Bourn," 7 Trewartha Park, Weston-super-Mare. |
| *Scarff, G., M.B., Ch.B., F.R.C.S. Ed. | 5 Rodney Place, Clifton. |
| *Scott, H. H. | 78 Old Market Street, Bristol. |
| *Shaw, J. E., M.B., C.M. Ed. | 23 Caledonia Place, Clifton. |
| *Shepherd, H. L., M.B., Ch.M. Brist. | 16 Victoria Square, Clifton. |
| *Short, A. R., M.D. | 69 Pembroke Road, Clifton. |
| *Short, L. J., M.D., B.S. Lond., M.D. Brist. | Maywood, Atlantic Road South, Weston-super-Mare. |
| *Smith, G. Cockburn, M.D. Brux. | 12 South Road, Newton Abbot. |

*Smith, T. Wilson ... ... ... ... ... ... ... ... 26 The Circus, Bath.
*Smith, W. Alexander, M.B., M.A. Oxon. ... 70 Pembroke Road, Clifton.
*Smyth, Richard, M.A., M.D. Dub. ... ... ... Burnley, Westbury-on-Trym, Bristol.
*Smythe, H. J. D., *M.C.*, M.B., M.S. ... ... 15 Eaton Crescent, Clifton.
*Somers, Mary, Miss, L.S.A. ... ... ... ... 2 Ashcombe Road, Weston-super-Mare.
*Spoor, A., M.B., B.Ch. Cantab. ... ... ... The Hydro, Bristol.
*Stanger, G. ... ... ... ... ... ... ... ... Olveston, Nr. Bristol.
*Staniland, M. F. ... ... ... ... ... ... ... 255 Stapleton Road, Bristol.
*Statham, R. S. S., *O.B.E.*, M.D., M.Ch. Brist. 10 Beaufort Road, Clifton, Bristol.
*Stewart, A. L., D.M., D.Ch. Brux. ... ... 31 Howard Road, Southville, Bristol.
*Stock, W. S. V., M.B. Lond. ... ... ... ... 1 Mortimer Road, Clifton.
*Strover, H. W. M., *O.B.E.*, M.B., Ch.B. Aberd. ... ... ... ... ... ... ... 24 Gloucester Road, Bishopston, Bristol.
*Sutton, G. E. F. ... ... ... ... ... ... ... 21 Victoria Square, Clifton.
*Symes, J. O., M.D. Lond. ... ... ... ... ... 71 Pembroke Road, Clifton.

*Tasker, D. G. C., M.B., M.S. Lond. ... ... 10 Christchurch Road, Clifton.
*Taylor, A. L., M.D. Lond.... ... ... ... ... Bristol General Hospital.
Taylor, A. L.... ... ... ... ... ... ... ... The Royal Infirmary, Bristol.
*Taylor, H. N., M.A. Cantab., M.D. Dub. ... Hillside, Ebbw Vale.
*Temple, G. H., M.B., C.M. Ed. ... ... ... Ailanthus, Weston-super-Mare.
*Terry, C. H., M.A., M.B., B.Ch. Oxon. ... ... 15 The Circus, Bath.
Thin, James ... ... ... ... ... ... ... ... 54 & 55 South Bridge, Edinburgh.
*Thompson, J., M.B., Ch.B. ... ... ... ... Eastbourne House, Devizes.
*Thomas, J. D., M.B. Cantab. ... ... ... ... Northwoods, Winterbourne.
*Todd, A. T., *O.B.E.*, M.B., Ch.B. Ed. ... ... 34 Alma Road, Clifton.
*Trist, J. R., R. *M.C.* ... ... ... ... ... 120 Henleaze Road, Clifton.
*Tryon, V., M.B., Ch.B. Brist. ... ... ... ... 3 Harley Place, Clifton.

*Vickery, W. H. ... ... ... ... ... ... ... 29 Trewartha Park, Weston-super-Mare.
*Vincent, A., M.B., Ch.B. ... ... ... ... ... 37 Chesterfield Road, St. Andrew's Park.

*Walker, Cyril H., M.B., B.A. Cantab. ... ... 8 Oakfield Road, Clifton.
*Wallace, John, *O.B.E.*, M.B., C.M. Ed. ... 10 Knightstone Road, Weston-super-Mare.
*Walters, C. F. ... ... ... ... ... ... ... 5 Mortimer Road, Clifton.
*Wanklyn-James, C. W., *O.B.E.* ... ... ... 3 Mortimer Road, Clifton.
*Warren, R., M.A., M.D. Oxon. ... ... ... Ellenborough Hall, Weston-super-Mare.
*Waterhouse, R., M.D. Lond. ... ... ... ... 25 The Circus, Bath.
*Watson-Williams, E., *M.C.*, B.A. Cantab. ... 12 Victoria Square, Clifton.
*Weston, B. A. A., M.B., B.Ch. Brist. ... ... 30 Cotham Grove, Bristol.
*Weatherhead, E., M.B., B.Ch. Cantab. ... 115 Queen's Road, Clifton, Bristol.
Weston, B. A., M.B. ... ... ... ... ... ... Brookside, Admaston, Wellington, Shropshire.
*Whelton, A., B.A., M.B., Ch.B. Cork ... ... Bristol Royal Infirmary.
*Whicher, A. H. ... ... ... ... ... ... ... 115 Queen's Road, Clifton.
*White, E. B. C. ... ... ... ... ... ... ... Mental Hospital, Fishponds, Bristol.
Whitehouse, H. Beckwith, M.S. Lond. ... 62 Hagley Road, Edgbaston, Birmingham.
Wigan, C. A., M.D. Durh., J.P. ... ... ... Deepdene, Portishead.
Wigmore, J., M.D., R.U.I. ... ... ... ... 16 Queen Square, Bath.
Willcox, R. Lewis ... ... ... ... ... ... 97 London Road, Salisbury.
Williams, S. R., M.B. Lond. ... ... ... ... Buckfastleigh, Devon.
*Wills, W. K., *O.B.E.*, M.B., B.C. Cantab. ... 1 Victoria Square, Clifton.
*Wood, Duncan ... ... ... ... ... ... ... 5 Eaton Crescent, Clifton.
*Wood, W. V., *M.C.*, B.A. Cantab. ... ... ... Henley Lodge, Yatton, Som.
*Wright, A. J., M.B., B.S. Lond. ... ... ... 2 Clifton Park, Clifton.

*Zealand, L., M.D. Man. ... ... ... ... ... Brecon Lodge, Westbury-on-Trym, Bristol.

